# The Influence of Hypocalcaemia on Trauma Patients Experiencing Post‐Induction Hypotension: A Retrospective Observational Study

**DOI:** 10.1111/1742-6723.70300

**Published:** 2026-06-12

**Authors:** Andy Kwok, Chris Selman, Kellie Gumm, David J. Read, Ned Douglas, Elyssia M. Bourke

**Affiliations:** ^1^ Department of Critical Care University of Melbourne Melbourne Australia; ^2^ Department of Emergency Medicine Royal Melbourne Hospital Melbourne Australia; ^3^ Clinical Epidemiology and Biostatistics Unit Murdoch Children's Research Institute Melbourne Australia; ^4^ Trauma Service Royal Melbourne Hospital Melbourne Australia; ^5^ Department of Surgery University of Melbourne Melbourne Australia; ^6^ Department of Anaesthesia Royal Melbourne Hospital Melbourne Australia; ^7^ Department of Emergency Medicine Grampians Health Ballarat Ballarat Australia

**Keywords:** airway, hypocalcaemia, induction agent, intubation, post‐induction hypotension

## Abstract

**Objective:**

Hypocalcaemia is a common electrolyte disturbance in major trauma patients, particularly those with haemorrhagic shock. Trauma patients are often intubated as part of their care, which can result in post‐induction hypotension. We aimed to estimate the effect of hypocalcaemia on post‐induction hypotension.

**Methods:**

We conducted a retrospective observational study of trauma patients ≥ 18 years old intubated in a tertiary Australian Emergency Department between 1st January 2020 and 30th September 2024. Epidemiological and clinical management data were extracted to estimate the effect of hypocalcaemia on outcomes.

**Results:**

Of 478 eligible patients, 151 had available haemodynamic and calcium measurements. Post‐induction hypotension occurred in 38/47 (81%) of hypocalcaemic and 69/104 (66%) of non‐hypocalcaemic patients. Hypocalcaemia was associated with higher odds of post‐induction hypotension (adjusted odds ratio [aOR] 2.15, 95% confidence interval [CI] 0.84–5.51), with considerable uncertainty in this estimate. Hypocalcaemic patients were more likely to receive red blood cells within 15‐min post‐induction (OR 2.13, 95% CI 1.12–4.04). Minimal differences were observed in cardiac arrest 15‐min post‐induction (OR 0.65, 95% CI 0.07–5.73) or mortality at discharge (OR 1.00, 95% CI 0.51–1.97).

**Conclusions:**

Hypocalcaemia may be associated with higher odds of post‐induction hypotension in trauma patients; however, our findings require corroboration across a larger multi‐centre prospective observational study. If corroborated, a randomised controlled trial is warranted to establish the utility of calcium replacement for the prevention of post‐induction hypotension.

## 1 Introduction

### 1.1 Background

Major trauma is a leading cause of emergency department (ED) presentations which may confer long‐term disability, reduced quality of life, and death [1]. Following injury, patients may experience airway obstruction, hypoxia, hypercapnia or cardiorespiratory arrest [2], mandating urgent endotracheal intubation. A potential complication of intubation is post‐induction hypotension; an event linked to impaired organ perfusion, acute kidney injury, myocardial injury, and prolonged hospitalisation [3]. Hypotension contributes to lactate driven metabolic acidosis [4] and coagulopathy, exacerbating the risk of haemorrhage [5]. Avoiding hypotension is crucial in patients with traumatic brain injuries, as even a single instance of hypotension can increase mortality [6]. However, the pathophysiology of post‐induction hypotension is not completely understood.

While risk factors such as age and injury severity have been identified [3], these are not modifiable. Existing strategies such as fluid boluses are linked to dilutional coagulopathy [7] and hyperchloremic metabolic acidosis [8], and vasopressors are usually avoided where a permissive hypotension approach is utilised [9, 10, 11]. Altering the dosage or choice of induction agents has had minimal impact on the incidence of post‐induction hypotension [12, 13]. Consequently, effective preventative interventions remain lacking. Calcium, due to its key role in blood pressure physiology [14], may represent a promising candidate.

Up to 56% of trauma patients experience at least one episode of hypocalcaemia [15]. Calcium has emerged as a promising therapeutic option for addressing both hypocalcaemia and haemodynamic instability in trauma patients [16, 17]. Restoring calcium levels may mitigate trauma‐related coagulopathy while enhancing contractility as a physiological inotrope through its role in excitation‐contraction coupling [14]. Calcium injections are already used to elevate blood pressure in other clinical settings including in paediatric patients [18] and in post‐operative hypotension [19, 20], demonstrating its potential efficacy and safety [14]. Despite existing literature investigating post‐induction hypotension and hypocalcaemia separately within trauma, evidence establishing the effect of hypocalcaemia on post‐induction hypotension in trauma patients intubated in the ED is lacking. Hence, in the present study we aim to establish the effect of hypocalcaemia on post‐induction hypotension for trauma patients being intubated in the ED setting.

## 2 Methods

### 2.1 Study Design and Setting

We conducted a single‐centre retrospective cohort study examining trauma patients aged ≥ 18 years old intubated in the Royal Melbourne Hospital (RMH) ED between 1st of January 2020 to 30th September 2024. The study protocol was approved by the RMH Human Research Ethics Committee (QA2024172).

### 2.2 Selection of Participants

We included adult patients aged ≥ 18 years who presented to the RMH ED following a traumatic injury and were intubated. We excluded patients < 18 years, patients intubated in either a pre‐hospital setting or outside of the ED (e.g., the operating theatre or intensive care unit), as well as patients intubated without the use of induction medications such as during cardiac arrest.

### 2.3 Data Collection

We extracted data from the RMH trauma registry, which collects patient demographics, clinical interventions, and outcome data [21]. Additional data not routinely collected by the trauma registry was abstracted from patient electronic medical records using a predefined data dictionary. Missing data were marked as unknown. Data were extracted by a single investigator (AK) after training with support from two others (EMB and ND) if uncertainty existed.

Systolic blood pressure was recorded at five predefined time points: immediately before, immediately after, 5, 10, and 15 min post‐induction, accepting a ±2‐min window. When multiple measurements existed within the time window, the measurement closest to the target time was selected.

Hypocalcaemia was assessed using ionised calcium results from arterial or venous blood gas samples measured in the ED on a point of care analyser (ABL90 FLEX PLUS, Radiometer Medical ApS, Brønshøj, Denmark). We defined hypocalcaemia as an ionised calcium level less than 1.12 mmol/L, which was the lower limit of the institution's reference range. If multiple calcium values were recorded, the calcium value immediately preceding the induction event was selected for analysis.

### 2.4 Outcomes

In this study, we defined the primary outcome as the incidence of post‐induction hypotension within 15 min following induction.

We defined post‐induction hypotension as a composite outcome of experiencing either absolute hypotension (systolic blood pressure < 90 mmHg) or relative hypotension (drop in systolic blood pressure ≥ 20% if pre‐induction systolic blood pressure was > 90 mmHg) within 15 min following induction. The additional criterion was included to capture changes in systolic blood pressure that did not meet the binary criterion of < 90 mmHg, but were clinically meaningful, especially in patients with traumatic brain injuries experiencing relative hypotension [22].

Secondary outcomes of this study included each component of the composite primary outcome, whether patients required interventions commonly used to treat hypotension (fluid bolus, blood product administration or vasopressors) within 15 min of induction, as well as patient outcomes including cardiac arrest within 15 min of induction and mortality at hospital discharge. We compared secondary outcomes across the different hypocalcaemia groups.

### 2.5 Statistical Analysis

We used convenience sampling, including records meeting all inclusion criteria and none of the exclusion criteria between 1st January 2020 to 30th September 2024. The statistical analysis plan was written with an anticipated end date of 31st December 2024. However, at database lock, data were only available to 30th September 2024, leading to a deviation from our pre‐specified analysis plan. No formal sample size calculation or power analysis was performed.

We summarised baseline characteristics using counts and percentages for categorical data and used means (standard deviations) for continuous variables unless the data were skewed, in which we reported the median and interquartile range (IQR).

The primary estimand of interest was an adjusted odds ratio between hypocalcaemic and non‐hypocalcaemic groups, which was estimated using a multivariate logistic regression model reported with its 95% confidence interval (CI) and *p‐*value. Directed acyclic graphs (DAGs) were used to identify variables to control for confounding (Figure S1) that were elicited from expert clinical opinion and existing literature. Details regarding induction agent choice and dosing in the overall cohort and stratified by hypocalcaemia status are provided in Tables S1 and S2.

Given anticipated missing data, missingness DAGs were used to define assumptions and guide handling of missingness (Figure S1). Based on these DAGs, missing data were addressed using multiple imputation with chained equations (50 datasets and 10 iterations between each imputation), including all variables in the analysis as well as auxiliary variables likely to influence missingness or the outcome [23]. Results were combined using Rubin's rules and reported with 95% CIs and *p* values. We conducted a sensitivity analysis by considering a scenario where we assumed patients with missing outcome data had a 1%, 5%, or 25% higher absolute risk of post‐induction hypotension than those with available data. We conducted this analysis using a delta‐adjusted approach (File S1). Complete case analyses were also performed to assess robustness.

Exploratory subgroup analyses for the primary outcome were conducted by injury severity score categories (1–11, 12–20, > 20), age (dichotomised as > 65 and ≤ 65 years), sex, and trauma mechanism (penetrating versus blunt injury). Each model was fitted within each subgroup separately.

For secondary outcomes, we analysed binary outcomes using logistic regression to estimate odds ratios (ORs) and continuous outcomes using mean differences reported with robust standard errors. All analyses were reported with corresponding 95% CIs and *p* values. Given the descriptive and exploratory nature of these analyses, no adjustments were made for potential confounders or for multiple comparisons. The observed associations should not be interpreted as evidence of causality. Instead, we focused on the magnitude and direction of effect estimates alongside their precision and considered the overall consistency of findings across outcomes. Analyses were conducted using Stata V.18.0 (Stata Corp, College Station, USA).

## 3 Results

### 3.1 Characteristics of the Study Subjects

**FIGURE 1:**
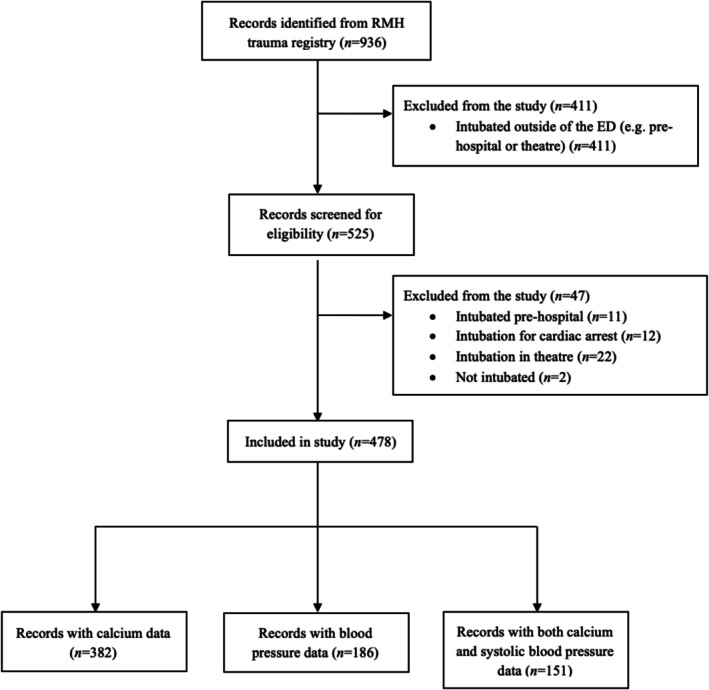
STROBE flow chart of participant selection. RMH, Royal Melbourne Hospital; ED, Emergency Department.

We identified 936 patients presenting to the RMH ED who were intubated during their hospital admission from January 1, 2020, to September 30, 2024, from the hospital trauma registry. We removed 458 patient records as they were either intubated in a pre‐hospital setting, intubated during cardiac arrest, intubated in theatre or intensive care unit, or not intubated at all. A total of 478 patients were included in the analysis (Figure 1). Baseline characteristics of participants are shown in Table 1. Our study sample had a mean age of 47 ± 20 years (SD) and was predominantly male (379/478, 79%). Most patients sustained blunt trauma (425/478, 89%). The median heart rate and systolic blood pressures in the hypocalcaemic group on arrival to the ED were 100 ± 24 (SD) and 123 ± 26 (SD) respectively, and 95 ± 26 (SD) and 135 ± 31 (SD) in the non‐hypocalcaemic group. The median injury severity score was 26 (IQR 18–35) in the hypocalcaemic group and 22 (IQR 13–29) in the non‐hypocalcaemic group.

**TABLE 1:** Baseline characteristics of eligible patients.

Patient characteristic	Hypocalcaemic (*n* = 100)	Non‐hypocalcaemic (*n* = 282)	Unknown calcium status (*n* = 96)	Study population (*n* = 478)
Age (years)				
Mean (SD)	48.7 (17.5)	46.9 (20.1)	47.2 (20.2)	47.4 (19.6)
Median [IQR]^a^	49.8 [35.2–60.3]	43.6 [29.3–61.7]	43.3 [30.1–61.2]	45.0 [30.3, 61.3]
Sex				
Male	77 (77%)	222 (79%)	80 (83.3%)	379 (79%)
Female	23 (23%)	60 (21%)	16 (16.7%)	99 (21%)
Pregnancy status, (*n* = 99)				
Not pregnant	22 (96%)	55 (92%)	15 (93.8%)	92 (93%)
Pregnant	1 (4%)	2 (3%)	0 (0.0%)	3 (3%)
Unknown	0 (0%)	3 (5%)	1 (6.2%)	4 (4%)
Mode of transport				
Self‐presenting	0 (0%)	1 (0%)	1 (1.0%)	2 (0%)
Ambulance (Road Car)	86 (86%)	241 (86%)	81 (84.4%)	408 (85%)
Air ambulance (Helicopter)	8 (8%)	19 (7%)	9 (9.4%)	36 (8%)
Ambulance vehicle but unknown type	4 (4%)	12 (4%)	1 (1.0%)	17 (4%)
Unknown	2 (2%)	9 (3%)	4 (4.2%)	15 (3%)
Pre‐hospital procedures^b^				
None	38 (38%)	132 (47%)	40 (41.7%)	210 (44%)
Chest decompression	11 (11%)	23 (8%)	12 (12.5%)	46 (10%)
Cardiopulmonary resuscitation	6 (6%)	8 (3%)	6 (6.2%)	20 (4%)
Pelvic binder	37 (37%)	90 (32%)	27 (28.1%)	154 (32%)
Intravenous fluids	39 (39%)	84 (30%)	37 (38.5%)	160 (34%)
Tourniquet	5 (5%)	12 (4%)	10 (10.4%)	27 (6%)
Other	10 (10%)	17 (6%)	10 (10.4%)	37 (8%)
Injury type				
Blunt	86 (86%)	253 (90%)	86 (89.6%)	425 (89%)
Penetrating	11 (11%)	25 (9%)	10 (10.4%)	46 (10%)
Burn	1 (1%)	3 (1%)	0 (0.0%)	4 (1%)
Unknown	2 (2%)	1 (0%)	0 (0.0%)	3 (1%)
Injury severity score (1–75)^a^	26.0 [18.0–34.5]	22.0 [13.0–29.0]	26.0 [14.0–33.0]	25.0 [14.0–30.0]
Vital signs on arrival to the emergency department				
Heart rate (bpm)	100.3 (23.6)	95.1 (25.5)	98.5 (28.8)	96.9 (25.9)
Systolic blood pressure (mmHg)	122.8 (26.3)	134.7 (30.7)	132.3 (33.9)	131.7 (30.8)
Respiratory rate (breaths/min)	20.6 (7.1)	19.6 (5.6)	19.5 (6.2)	19.8 (6.0)
Oxygen saturation (%)^a^	98.0 [94.0–100.0]	99.0 [96.0–100.0]	99.0 [95.0–100.0]	99.0 [95.0–100.0]
Glasgow Coma scale (3–15)^a^	11.0 [7.0–14.0]	10.0 [6.0–13.0]	7.0 [3.0–13.0]	10.0 [5.0–13.0]
Vasopressor exposure before induction^b^				
None	87 (87%)	257 (91%)	88 (92%)	432 (90%)
Adrenaline	4 (4%)	7 (3%)	3 (3%)	14 (3%)
Metaraminol	8 (8%)	19 (7%)	6 (6%)	33 (7%)
Noradrenaline	1 (1%)	6 (2%)	1 (1%)	8 (2%)
Blood product requirement before induction^b^				
Red blood cells	37 (37%)	59 (21%)	21 (22%)	117 (25%)
Fresh frozen plasma	17 (17%)	14 (5%)	3 (3%)	34 (7%)
Cryoprecipitate	0 (0%)	1 (0%)	0 (0%)	1 (0%)
Platelets	10 (10%)	8 (3%)	0 (0%)	18 (4%)
None	62 (62%)	223 (79%)	75 (78%)	360 (75%)

*Note:* Missing data noted for: Heart rate (*n* = 2), systolic blood pressure (*n* = 3), respiratory rate (*n* = 22), oxygen saturation (*n* = 8), GCS (*n* = 4), and baseline calcium level (*n* = 96).

^a^ Skewed distribution represented as median [IQR].

^b^ Total percentages may exceed 100% as patients could receive more than one intervention.

### 3.2 Main Results

Primary outcome data (Table 2) were available for 151/478 (32%) patients with both ionised calcium levels and at least one blood pressure recording at a predefined time point (immediately post‐induction or at 5‐, 10‐, or 15‐min post‐induction). Although 186 patients had post‐induction blood pressure data available, not all had corresponding pre‐induction ionised calcium measurements recorded within the study timeframe, resulting in 151 patients being included in the primary analysis cohort. Hypocalcaemic trauma patients had increased odds of developing post‐induction hypotension compared to non‐hypocalcaemic patients (aOR 2.15, 95% CI 0.84–5.51; *p* = 0.11). However, the wide CI indicates substantial uncertainty, with estimates compatible with both a clinically meaningful effect and no effect. Across most subgroups, including age, sex, and injury type, there was little evidence of effect modification in the association between hypocalcaemia and post‐induction hypotension. An exception was patients with an injury severity score > 20, where 6/9 (91%) of hypocalcaemic patients versus 11/21 (76%) of non‐hypocalcaemic patients experienced post‐induction hypotension (OR 8.64; 95% CI 1.31–56.93; *p* = 0.03). All hypocalcaemic patients aged > 65 years old (*n* = 13) experienced post‐induction hypotension, although the small sample size limits inference. However, these subgroup analyses were based on very small numbers, resulting in imprecise and potentially unstable estimates given by the width of the 95% CIs. While the direction of effect was consistent, the magnitude varied considerably across subgroups; therefore, these findings should be regarded as hypothesis‐generating only.

**TABLE 2:** Primary outcome: Association between baseline hypocalcaemia and post‐induction hypotension.

	Hypocalcaemic, *n*/*N* (%)	Non‐hypocalcaemic, *n*/*N* (%)	Unadjusted OR (95% CI)	*p*	Adjusted marginal OR (95% CI)	*p*
Primary analysis (complete case)	38/47 (81%)	69/104 (66%)	2.14 (0.93, 4.93)	0.07	1.86 (0.65, 5.36)	0.25
Primary analysis (multiple imputation)^a^			2.51 (1.20, 5.23)	0.02	2.15 (0.84, 5.51)	0.11
Subgroup analysis						
Injury severity score						
1–11	1/4 (25%)	13/24 (54%)	Not estimable^b^	NA	NA	NA
12–20	6/9 (67%)	11/21 (52%)	2.26 (0.19, 27.58)	0.52	NA	NA
20+	31/34 (91%)	45/59 (76%)	8.64 (1.31, 56.93)	0.03	NA	NA
Age						
> 65 years	13/13 (100%)	25/35 (71%)	Not estimable^b^	NA	NA	NA
65 years and less	25/34 (74%)	44/69 (64%)	1.74 (0.58, 5.22)	0.32	NA	NA
Sex						
Male	28/35 (80%)	51/80 (64%)	2.84 (0.80, 10.16)	0.11	NA	NA
Female	10/12 (83%)	18/24 (75%)	3.22 (0.11, 94.97)	0.50	NA	NA
Injury type						
Penetrating injury	5/6 (83%)	6/9 (67%)	Not estimable^b^	NA	NA	NA
Blunt injury	32/40 (80%)	63/95 (66%)	1.84 (0.59, 5.75)	0.29	NA	NA

*Note:* Adjusted odds ratios were estimated using logistic regression, adjusting for pre‐induction lactate levels, pre‐induction calcium administration, pre‐hospital blood product use, shock index, induction agent, vasopressor use, fluid bolus administration, age (> 65 vs. ≤ 65), and injury severity score category (one–11, 12–20, > 20). Effect modifiers were considered, but models did not converge due to limited events. Subgroup analyses were not adjusted for confounding variables and are intended to be purely hypothesis generating.

Abbreviations: CI, confidence interval; NA, not applicable; OR, odds ratio.

^a^ Multiple imputation performed under the assumption of data missing at random.

^b^ Not estimable indicates that the odds ratio could not be computed due to insufficient data.

Secondary outcomes are summarised in Table 3. Hypocalcaemia was associated with higher odds of the patient experiencing a systolic blood pressure < 90 mmHg within 15 min post‐induction (OR 2.27; 95% CI 1.19–4.34). In contrast, there was little evidence of an association between hypocalcaemia and a systolic blood pressure drop of ≥ 20% anytime 15 min post‐induction, if the initial systolic blood pressure before induction was > 90 mmHg (OR 1.20; 95% CI 0.60–2.37). Fluid bolus volumes did not differ between groups on average (MD 3.76 mL; −61.52–69.04 mL). Hypocalcaemic patients were associated with having higher odds of receiving blood products within 15 min post‐induction, particularly red blood cells (OR 2.13; 95% CI 1.12–4.04). They also received a greater volume of blood products overall on average (MD 80.24 mL; 95% CI 3.28–157.19 mL). There was little evidence of differences in patient outcomes between the two groups including cardiac arrest within the first 15 min post‐induction (OR 0.65; 95% CI 0.07–5.73) or mortality at hospital discharge (OR 1.00; 95% CI 0.51–1.97).

**TABLE 3:** Secondary outcomes within 15 min post‐induction and at hospital discharge using multiple imputation to handle missing data.

	Hypocalcaemia (*n* = 100)	Non‐hypocalcaemia (*n* = 282)	Complete case analysis		Multiple imputation	
			Unadjusted odds ratio (95% CI)	*p*	Unadjusted odds ratio (95% CI)	*p*
Systolic blood pressure < 90 mmHg at any time 15 min post‐induction	22/41 (54%)	32/84 (38%)	1.88 (0.88, 4.01)	0.10	2.27 (1.19, 4.34)	0.01
≥ 20% decrease in systolic blood pressure any time 15 min post‐induction if pre‐induction systolic blood pressure was > 90 mmHg	26/43 (61%)	57/99 (58%)	1.13 (0.54, 2.34)	0.75	1.20 (0.60, 2.37)	0.61
Fluid bolus requirements within 15 min post‐induction	7/100 (7%)	20/282 (7%)	0.99 (0.40, 2.41)	0.98	0.93 (0.39, 2.24)	0.87
Blood product requirements within 15 min post‐induction						
Red blood cells	19/100 (19%)	28/282 (10%)	2.13 (1.13, 4.01)	0.02	2.13 (1.12, 4.04)	0.02
Fresh frozen plasma	12/100 (12%)	18/282 (6%)	2.00 (0.93, 4.32)	0.08	1.97 (0.91, 4.28)	0.09
Platelets	8/100 (8%)	11/282 (4%)	2.14 (0.84, 5.49)	0.11	2.21 (0.85, 5.74)	0.10
Vasopressor requirement within 15 min post‐induction	29/100 (29%)	63/282 (22%)	1.42 (0.85, 2.38)	0.18	0.74 (0.44, 1.24)	0.25
Cardiac arrest within 15 min post‐induction	1/100 (1%)	4/282 (1%)	0.70 (0.08, 6.36)	0.75	0.65 (0.07, 5.73)	0.69
Mortality at discharge	15/100 (15%)	40/282 (14%)	1.07 (0.56, 2.03)	0.84	1.00 (0.51, 1.97)	0.99

	Hypocalcaemia (*n* = 100)	Non‐hypocalcaemia (*n* = 282)	Complete case analysis		Multiple imputation	
			Mean difference (95% CI)	*p*	Mean difference (95% CI)	*p*
Total volume of fluid bolus within 15 min post‐induction (mL)	71.0 (293.10)	64.2 (241.20)	6.82 (−57.25, 70.88)	0.83	3.76 (−61.52, 69.04)	0.91
Percentage reduction of systolic blood pressure from prior, induction, 15 min post‐induction (%)	−9.9 (14.60)	−0.4 (26.10)	−9.51 (−16.95, −2.06)	0.01	−4.51 (−11.60, 2.59)	0.21
Total volume of all blood products within 15 min post‐induction (mL)	134.9 (307.50)	65.0 (181.80)	69.91 (5.94, 133.89)	0.03	80.24 (3.28, 157.19)	0.04

*Note:* Analyses were exploratory and unadjusted due to limited events. Positive odds ratios indicate higher odds of the outcome in hypocalcaemic patients; negative median differences indicate lower values compared to normocalcaemic or hypercalcaemic patients.

Abbreviation: CI, Confidence interval.

## 4 Discussion

To our knowledge, this study is the first to evaluate the direct relationship between hypocalcaemia and post‐induction hypotension in trauma patients. Our findings suggest hypocalcaemic trauma patients may have higher odds of developing post‐induction hypotension, particularly with an injury severity score > 20. This effect size should be interpreted cautiously given the uncertainty around the effect estimate. Hypocalcaemic patients received more blood products, possibly because clinicians viewed the hypotension as clinically significant and took steps to mitigate it. Alternatively, the hypocalcaemic group may have been more unwell and required more blood products, suggesting that hypocalcaemia is a marker of traumatic injury rather than causally contributing to hypotension.

This study proposes hypocalcaemia as a potentially modifiable risk factor in the trauma population, without being able to definitively resolve whether hypocalcaemia causes post‐induction hypotension. While this idea has existed in clinical practice for some time, the translation of this physiological understanding to trauma resuscitation has been limited by a lack of evidence linking hypocalcaemia to haemodynamic instability during induction. While the observed effect is biologically consistent with the established pathophysiological role of calcium in cardiac contractility as hypocalcaemia impairs myocardial function through impaired excitation‐contraction coupling [14], a more robust and complete prospective study is likely required to answer this question. We believe our data supports several of the Bradford‐Hill criteria for causality, including the strength of the association, the temporal relationship with hypocalcaemia being measured before hypotension, and the plausibility and coherence of hypocalcaemia influencing blood pressure. In our view, hypocalcaemia most likely moderates the effect of induction drugs on blood pressure, rather than being a downstream biomarker that is an outcome of induction drug administration. However, fully resolving this will require either intervening on calcium (i.e., a randomised controlled trial) or a carefully designed prospective observational study.

An interesting finding was that after separating our composite outcome into two individual secondary outcomes, hypocalcaemic patients were more likely to experience absolute hypotension (systolic blood pressure < 90 mmHg), but not relative hypotension. Future research should consider whether a binary threshold is the optimal definition of post‐induction hypotension. Ideally, this would involve data linking both absolute and relative thresholds to patient outcomes, interventional studies, and an international consensus being developed to harmonise the primary outcomes of future research.

In our study, 71% of the patients experienced post‐induction hypotension, irrespective of their hypocalcaemia status. This exceeds rates of up to 44% reported in prior retrospective studies [24]. We hypothesise this difference likely reflects population characteristics. As a major trauma centre, RMH preferentially receives the most critically ill trauma patients who are at high risk of haemodynamic deterioration. This contrasts with the ED cohort in Green et al., which was drawn from a centre not designated as a Level One trauma centre. Our cohort therefore likely represents greater injury severity and haemodynamic vulnerability, potentially explaining the higher observed incidence.

The subgroup analysis in patients with an injury severity score > 20 suggested hypocalcaemic trauma patients have over eight times the odds of developing post‐induction hypotension compared to non‐hypocalcaemic trauma patients. This association is biologically plausible as severely injured patients likely have greater physiological stress, more pronounced calcium derangements from massive blood transfusions, and reduced compensatory reserves [25]. Our findings identify the highest risk population where calcium monitoring and correction may provide the greatest benefit. We acknowledge that these findings are uncertain given the limited number of patients in these subgroups, and further work is required to corroborate these findings.

Current trauma guidelines emphasise early identification and correction of coagulopathy [26]. Yet, calcium monitoring remains underutilised [27] despite its role in both haemodynamic stability and the coagulation cascade [28]. While ionised calcium measurements are routinely obtained through blood gas tests in trauma patients presenting to EDs, calcium results are not integrated into pre‐induction assessment protocols, nor is calcium routinely replaced in this setting [27]. Our findings suggest that incorporating calcium status may help mitigate the incidence of post‐induction hypotension.

This study has many strengths, including a representative sample of adult patients with a significant burden of traumatic injury, the use of real‐world observed data, and a focus on clinically important outcomes that are relevant to patients and clinicians. These results provide an early signal supporting a large multi‐centre prospective observational study to better understand whether hypocalcaemia is causally associated with post‐induction hypotension. There are several limitations. We had an extensive amount of missing data due to using records originating from clinical care in critically unwell patients. We attempted to mitigate the impact of this by employing advanced biostatistical methods to handle the missing data, but a more complete dataset may be required to achieve high confidence in the associations we found. In addition, despite adjusting for confounders in the primary analysis model, there is potential for residual confounding in unmeasured confounders. Post‐induction hypotension was determined using episodic blood pressure recordings, rather than through continuous monitoring. Transient or immediate hypotensive episodes occurring outside these time points may not have been captured. While continuous invasive arterial blood pressure monitoring would provide more precise haemodynamic assessment, it is often not feasible during the early acute resuscitation phase of trauma reception. Given that this was a single centre conducted in a major trauma centre receiving a highly selected cohort of severely injured patients, our findings may have limited generalisability to broader ED populations with lower acuity cases. Finally, our study was restricted to short‐term clinical outcomes. While these outcomes are important in a trauma setting, we have no data to reflect functional recovery or quality of life. Consumer involvement will be crucial to determining important endpoints from a patient perspective.

## 5 Conclusions

Adult hypocalcaemic trauma patients may be associated with higher estimated odds of post‐induction hypotension and greater blood product use following intubation compared to their non‐hypocalcaemic counterparts. However, these findings warrant corroboration with further prospective studies to clarify these relationships. If corroborated, a randomised controlled trial of calcium supplementation prior to intubation would be useful to establish whether correction of hypocalcaemia can reduce the incidence of post‐induction hypotension in trauma patients. These findings did not translate into differences in the risk of peri‐intubation cardiac arrest or hospital mortality; however, the study was likely underpowered to detect meaningful differences in these rare outcomes.

## Author Contributions

A.K., N.D. and E.M.B. conceived the study. E.M.B. obtained ethics and governance approval. A.K. and K.G. obtained the data. C.S. cleaned and analysed the data. A.K., C.S., N.D., and E.M.B. interpreted the data. A.K. drafted the initial manuscript, with all authors contributing substantially to its revision. E.M.B. takes responsibility for the paper as a whole.

## Funding

The authors have nothing to report.

## Conflicts of Interest

The authors declare no conflicts of interest.

## Supporting information


**Figure S1:** Directed acyclic graph of confounding variables influencing hypocalcaemia and post‐induction hypotension.
**Table S1:** Induction agents and corresponding induction doses administered during endotracheal intubation.
**Table S2:** Induction agents and corresponding induction doses administered during endotracheal intubation by hypocalcaemia status.
**File S1:** Statistical analysis plan.

## Data Availability

The study data are held at the Royal Melbourne Hospital in Melbourne, Australia. Requests for data can be made at any time and can be initiated by email to elyssia.bourke3@mh.org.au. Requests will be considered within existing administrative, ethical and regulatory constraints and ongoing research by the study team.
