# Supplementary material for: The Influence of Hypocalcaemia on Trauma Patients Experiencing Post‐Induction Hypotension: A Retrospective Observational Study
Source: Emerg Med Australas. 2026 Jun 12;38(3):e70300. doi: 10.1111/1742-6723.70300 (PMC13263128; doi:10.1111/1742-6723.70300)

**Supplementary Appendix**

Supplement to: Kwok et al.

*The Influence of Hypocalcaemia on Trauma Patients Experiencing Post-Induction Hypotension: A Retrospective Observational Study*

This appendix has been provided by the authors to give readers additional information about the work

**Table of Contents**

| Figure S1. Directed Acyclic Graph of Confounding Variables Influencing Hypocalcaemia and Post-Induction Hypotension | Page 3 |
| --- | --- |
| Table S1. Induction Agents and Corresponding Induction Doses Administered During Endotracheal Intubation | Page 4 |
| Table S2. Induction Agents and Corresponding Induction Doses Administered During Endotracheal Intubation by Hypocalcaemia Status | Page 5 |
| Supplementary File 1. Statistical Analysis Plan | Page 6 |

**Figure S1.** Directed Acyclic Graph of Confounding Variables Influencing Hypocalcaemia and Post-Induction Hypotension

*
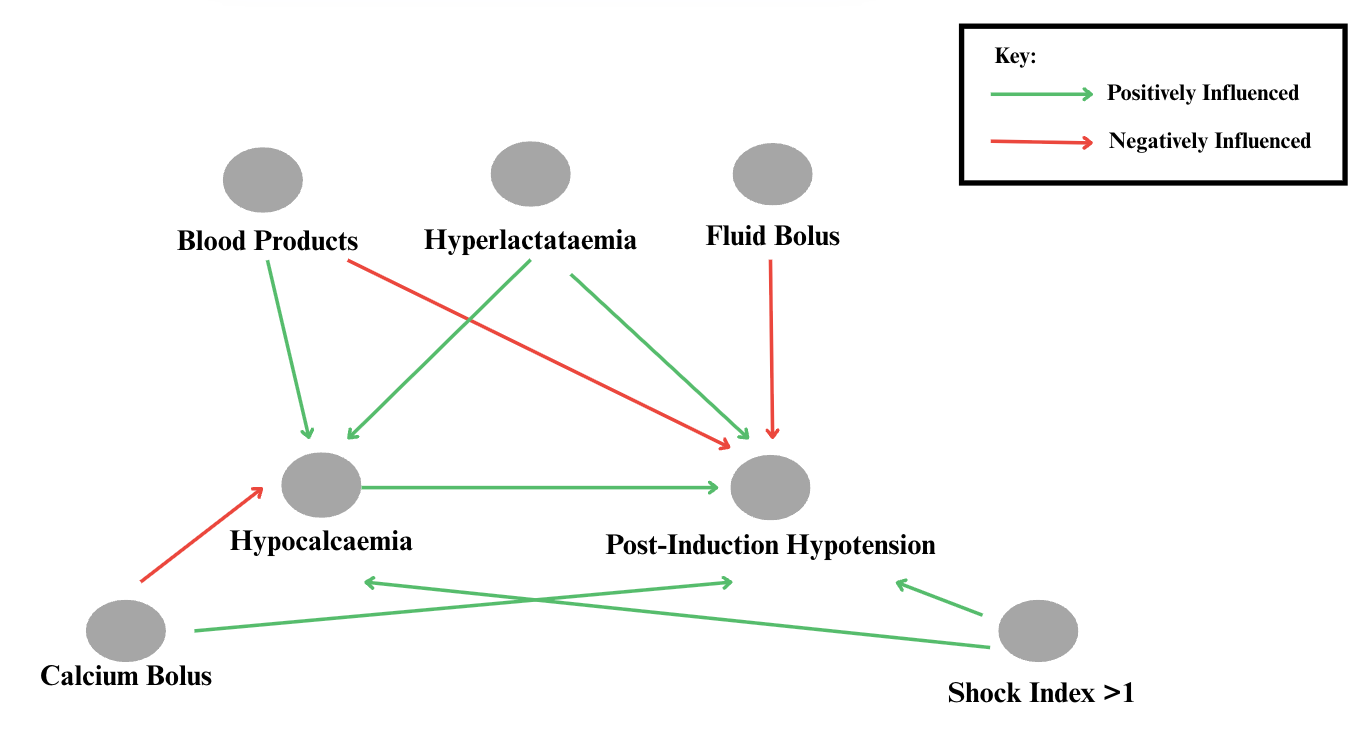
*Green arrows (→) represent positive influences where an increase in the source factor leads to an increase in the target factor. Red arrows (→) represent negative influences where an increase in the source factor leads to a decrease in the target factor. Hypocalcaemia is defined as an ionized calcium level < 1.12 mmol/L. Hyperlactatemia is defined as a lactate level ≥ 4 mmol/L. Shock index is calculated as the heart rate in beats per minute divided by the systolic blood pressure in mmHg.

**Table S1.** Induction Agents and Corresponding Induction Doses Administered During Endotracheal Intubation

|  | **n (%) or Median (IQR)  (N=478)** |
| --- | --- |
| **Induction Agents Used^†^** |  |
| Fentanyl | 158 (33%) |
| Ketamine | 313 (66%) |
| Midazolam | 36 (8%) |
| Propofol | 123 (26%) |
| **Dosing of Induction Agent Used^†^** |  |
| Fentanyl Dose (mcg) | Median = 100.0 (IQR = 50.0, 100.0) |
| Ketamine Dose (mg) | Median = 100.0 (IQR = 50.0, 100.0) |
| Midazolam Dose (mg) | Median = 3.0 (IQR = 2.0, 5.0) |
| Propofol Dose (mg) | Median = 80.0 (IQR = 50.0, 100.0) |

Induction agents administered during endotracheal intubation and corresponding induction doses among the study cohort (N = 478). Data are presented as n (%) or median (IQR).

^†^ More than one induction agent could have been administered to the same patient and hence the total count will be larger than the total sample size of the study.

**Table S2.** Induction Agents and Corresponding Induction Doses Administered During Endotracheal Intubation by Hypocalcaemia Status

|  | **Hypocalcaemic (n=100)** | **Non-Hypocalcaemic (n=282)** |
| --- | --- | --- |
| **Induction Agents Used^†^** |  |  |
| Fentanyl | 36 (36%) | 95 (34%) |
| Ketamine | 63 (63%) | 184 (65%) |
| Midazolam | 7 (7%) | 22 (8%) |
| Propofol | 26 (26%) | 73 (26%) |
| **Dosing of Induction Agent Used** |  |  |
| Fentanyl Dose (mcg) | 100 [50-150] | 100 [50-125] |
| Ketamine Dose (mg) | 80 [50-100] | 100 [60-120] |
| Midazolam Dose (mg) | 2 [1-5] | 3 [2-5] |
| Propofol Dose (mg) | 90 [50-100] | 80 [50-100] |

Induction agents administered during endotracheal intubation and corresponding induction doses among hypocalcaemic (n=100) and non-hypocalcaemic (n=282) patients. Data are presented as n (%) or median [IQR].

^†^ More than one induction agent could have been administered to the same patient and hence the total count will be larger than the total sample size of the study.

**Supplementary File 1.** Statistical Analysis Plan
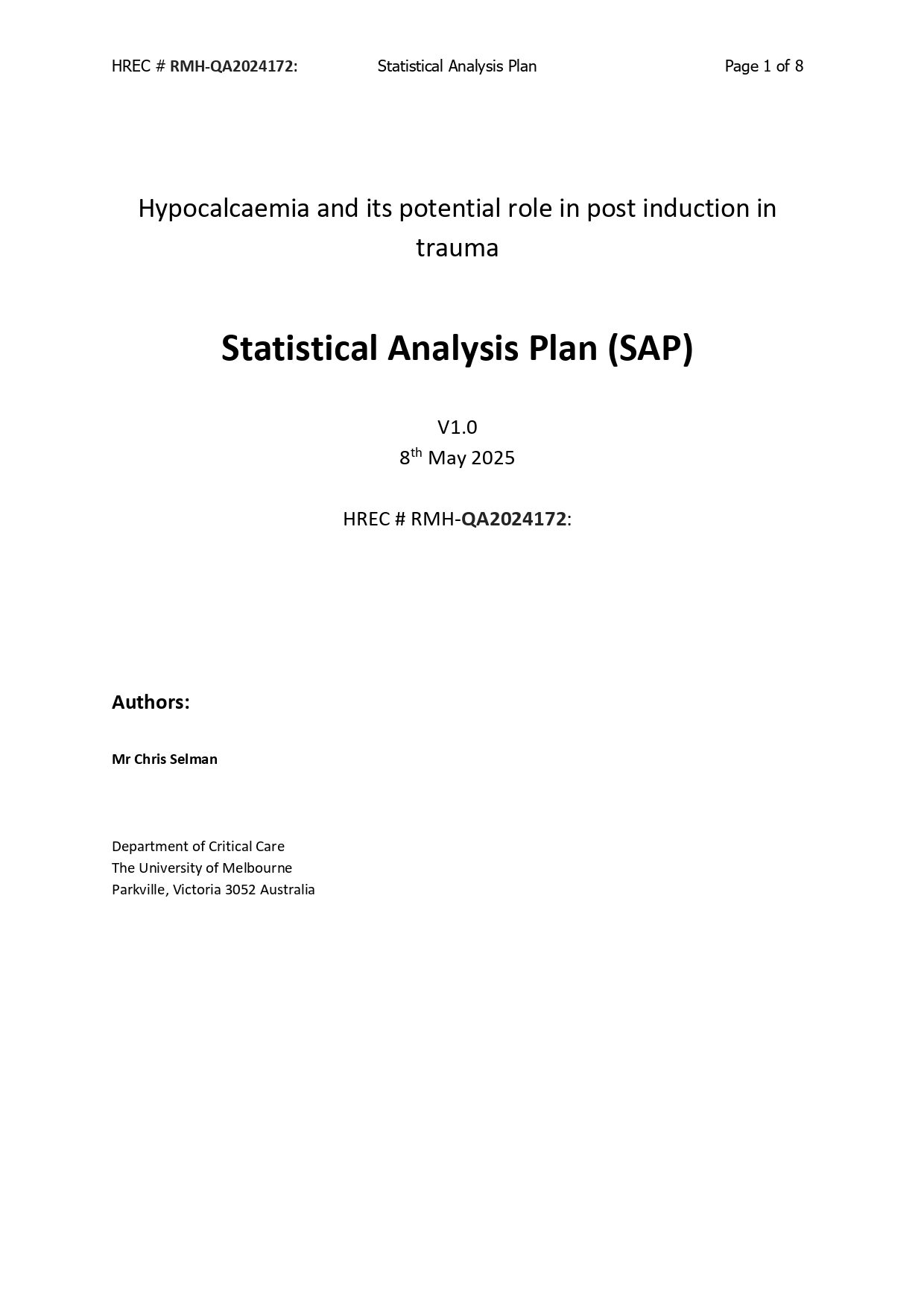


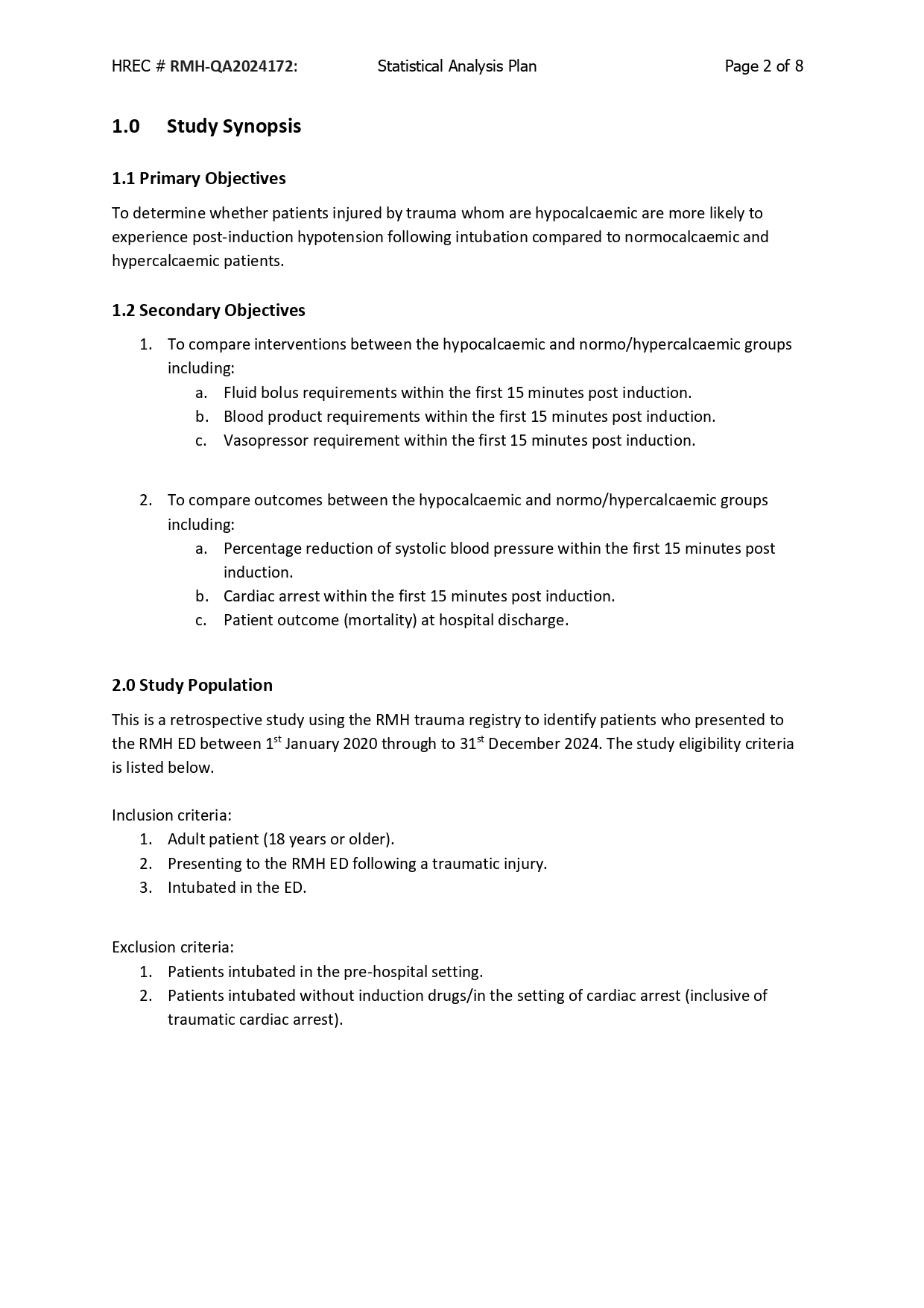

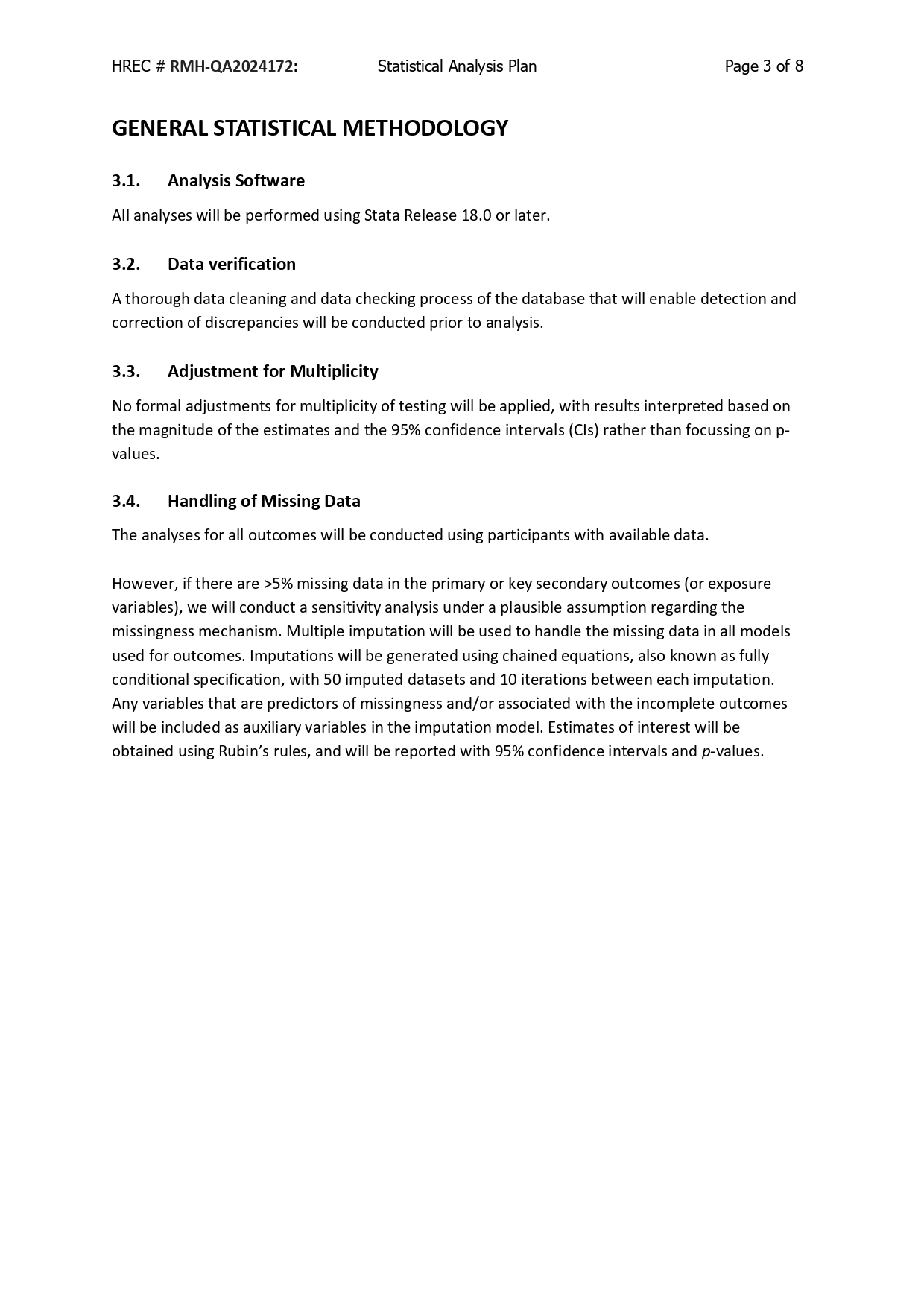

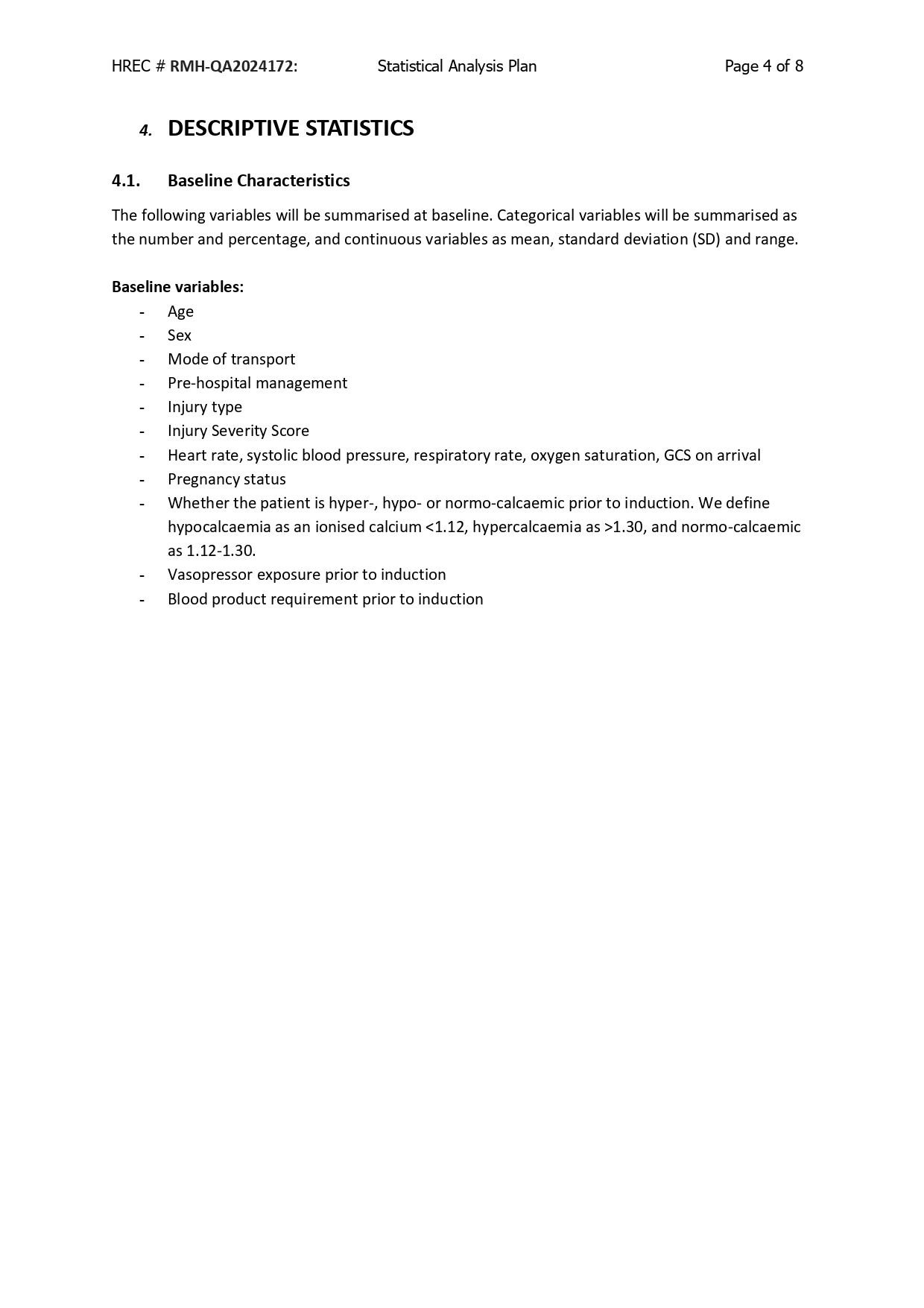

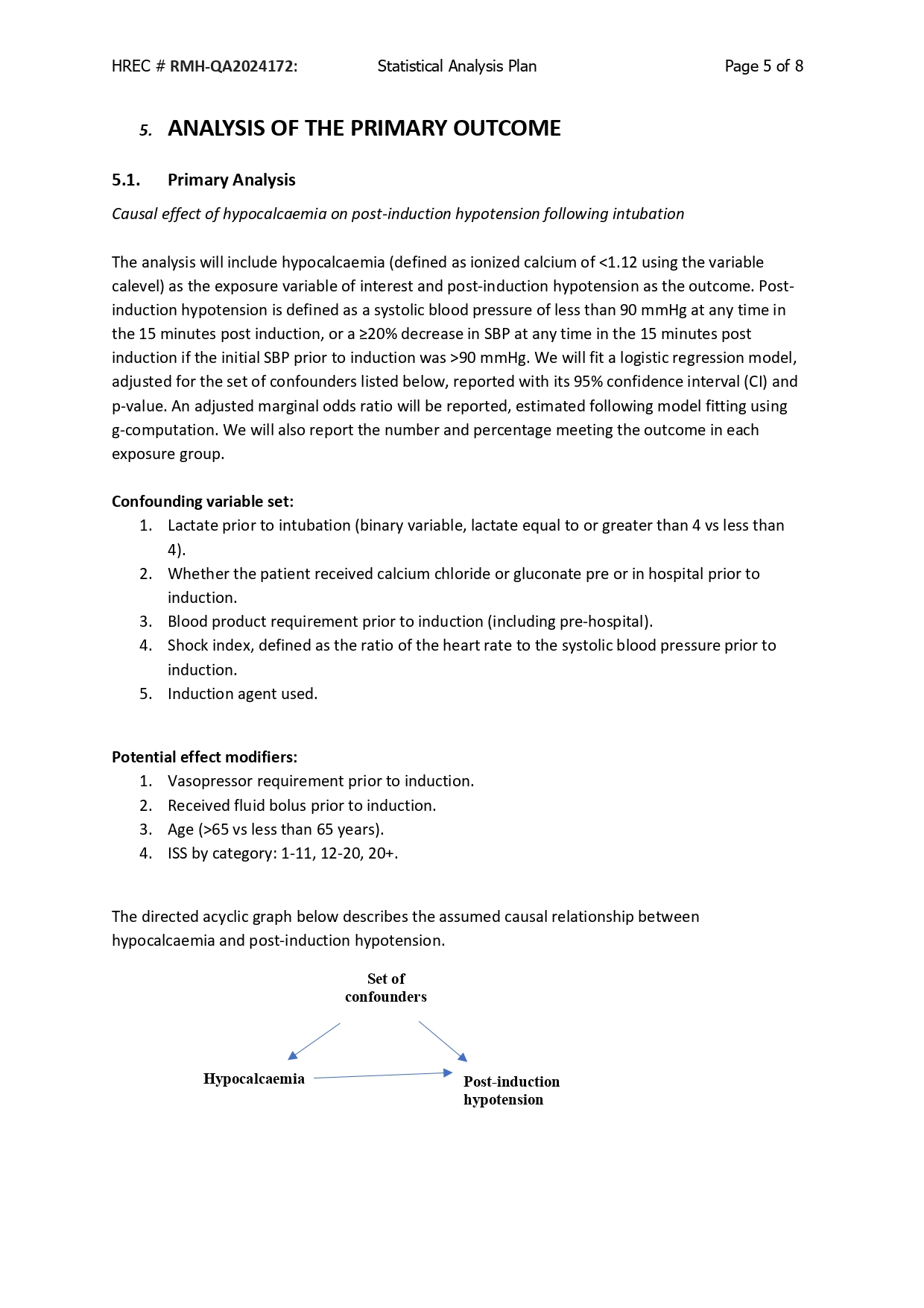

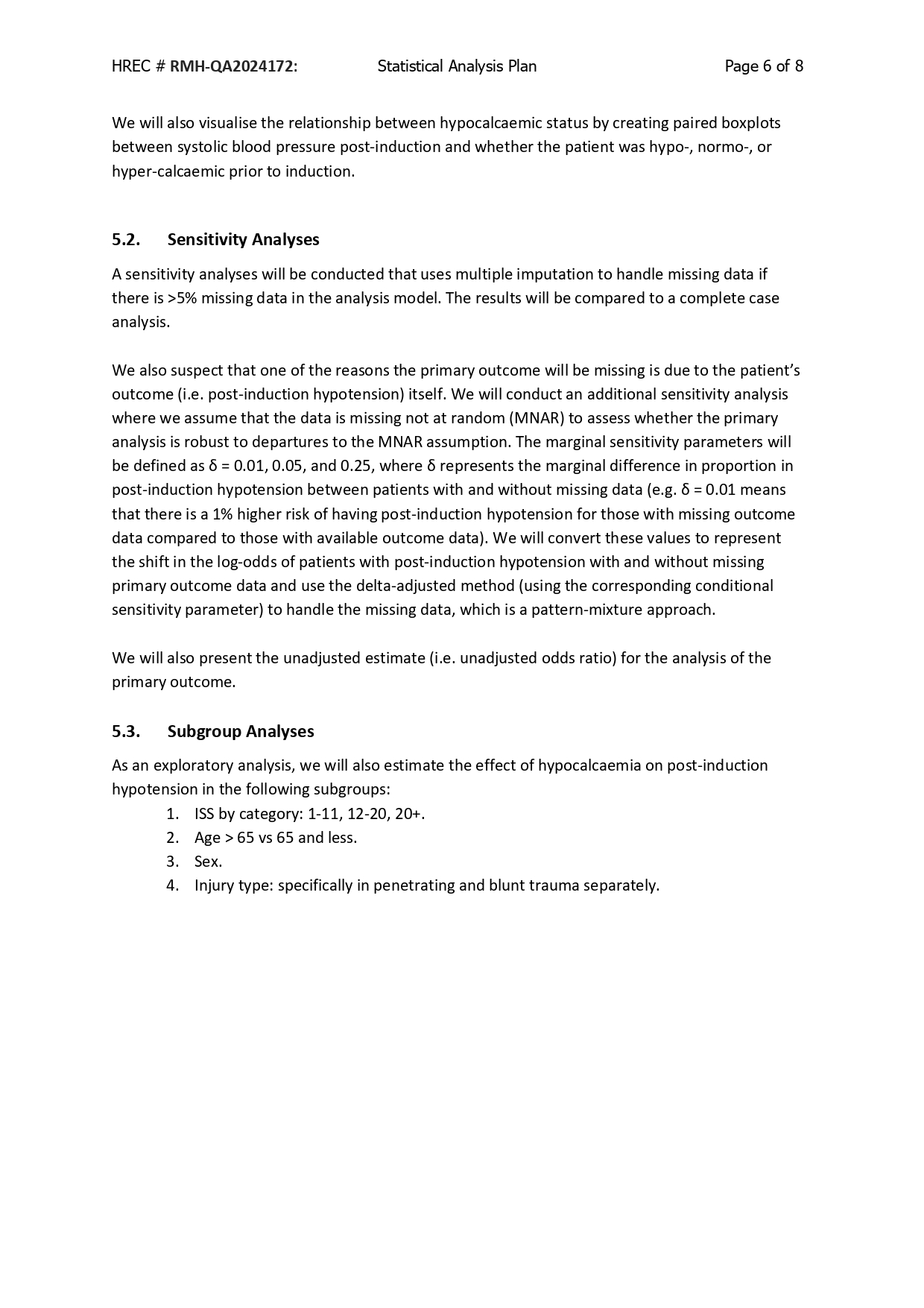

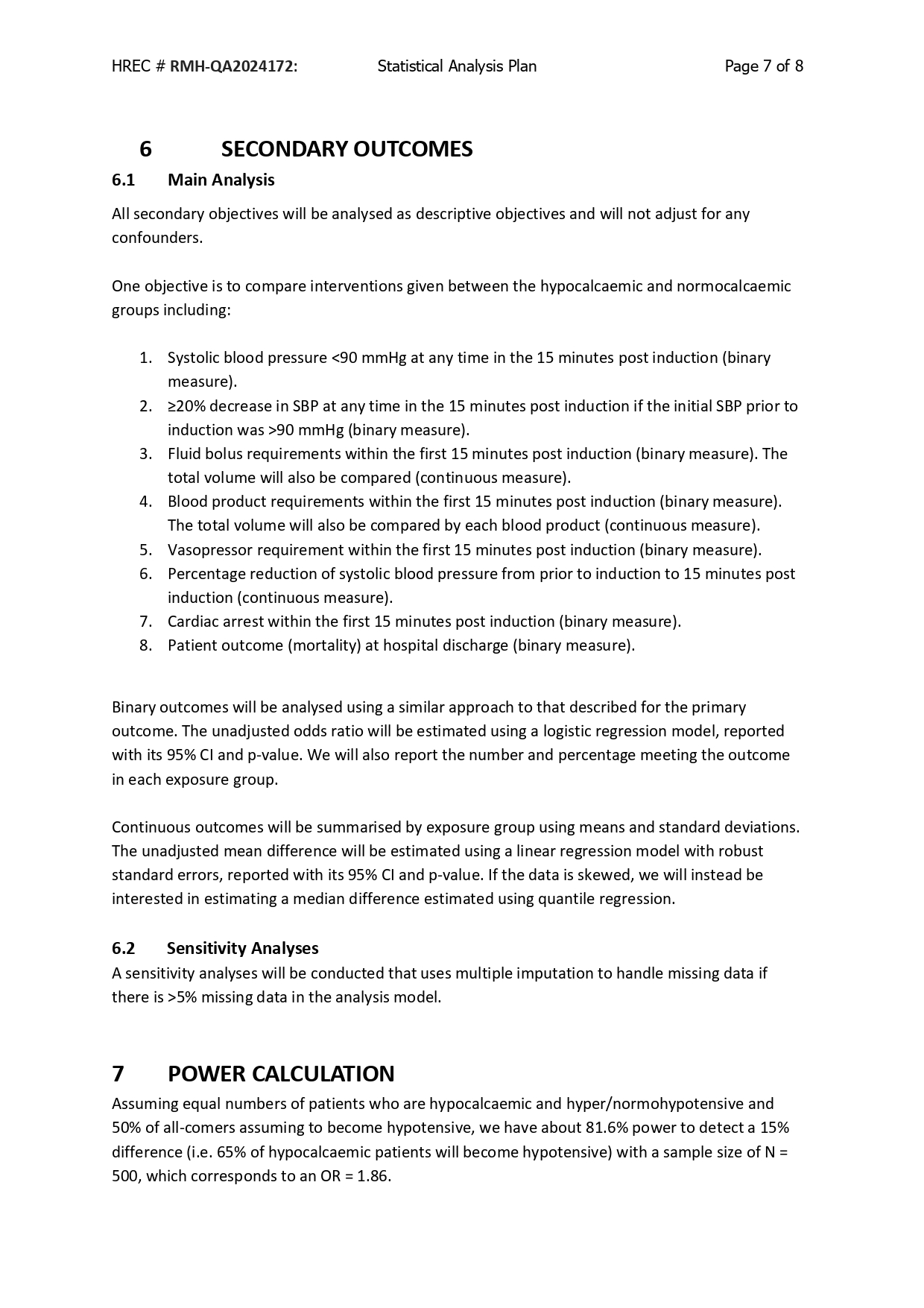

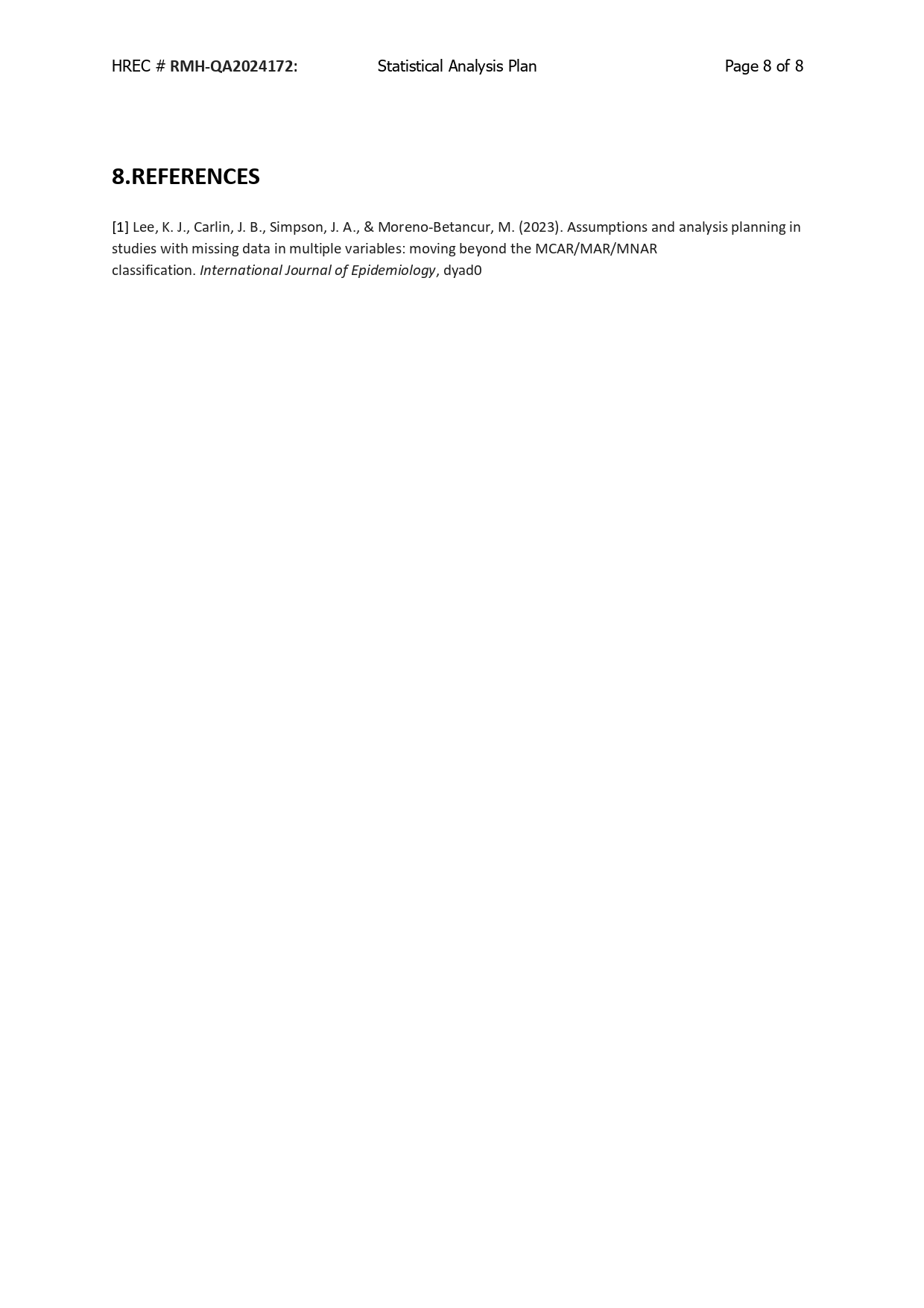

Supplement: Supplementary file 1 — Figure S1: Directed acyclic graph of confounding variables influencing hypocalcaemia and post‐induction hypotension. Table S1: Induction agents and corresponding induction doses administered during endotracheal intubation. Table S2: Induction agents and corresponding induction doses administered during endotracheal intubation by hypocalcaemia status. File S1: Statistical analysis plan. [file EMM-38-0-s001.docx]
